
JOURNAL INFORMATION
==============================
NLM Title Abbreviation: Wirtschaftsdienst
Journal ID: Wirtschaftsdienst
NLM Journal ID: 101086612

Wirtschaftsdienst (Hamburg, Germany : 1949)

ISSN: 0043-6275

ARTICLE INFORMATION
==============================
PMCID: PMC7765696
PMID: 33390626
DOI: 10.1007/s10273-020-2808-y
Article ID: 2808
Article version: 1
Subjects: Ökonomische Trends

Konjunkturschlaglicht: Coronavirus beherrscht die deutsche Wirtschaft

Coronavirus dominates the German economy

Hinze Jörg hinze@hwwi.org

grid.435054.3 0000 0001 2227 8589 HWWI, Oberhafenstraße 1, 20097 Hamburg, Deutschland
Electronic publication date: 2020 Dec 26
Print publication date: 2020
Volume: 100
Issue: 12
First page: 979
Last page: 980
Copyright: © Der/die Autor(en) 2020
License: Dieser Artikel wird unter der Creative Commons Namensnennung 4.0 International Lizenz (https://creativecommons.org/licenses/by/4.0/deed.de) veröffentlicht.
Open Access wird durch die ZBW — Leibniz-Informationszentrum Wirtschaft gefördert.
License URL: https://creativecommons.org/licenses/by/4.0/

issue-copyright-statement: © The Author(s) 2020

==============================
DOI: 10.1007/s10273-020-2808-y

### Konjunkturschlaglicht

### Coronavirus beherrscht die deutsche Wirtschaft

Die Entwicklung der deutschen Wirtschaft wird seit Anfang dieses Jahres von der Ausbreitung der Corona-Pandemie bestimmt. Diese Krise ist, wenngleich die Industrie sich damals schon im Abschwung befand, weniger konjunkturell als vielmehr durch externe Faktoren bedingt, nämlich die Corona-Epidemie und dadurch ausgelöste politische Entscheidungen, die Lockdowns. Nach dem ersten Lockdown im Frühjahr verzeichnete die deutsche Wirtschaft in der ersten Hälfte dieses Jahres einen massiven Einbruch; das reale, saisonbereinigte Bruttoinlandsprodukt (BIP) ging in den ersten beiden Quartalen (jeweils gegenüber dem Vorquartal) um 2 % bzw. knapp 10 % zurück. Zwischenzeitlich hat sich die deutsche Wirtschaft wieder deutlich erholt. Im dritten Quartal ist das reale BIP saisonbereinigt um 8½ % gestiegen (vgl. Abbildung 1).

Seit Herbstbeginn ist jedoch eine zweite Corona-Welle im Gange; die Infektionszahlen der vergangenen Wochen sind erheblich höher als die im Frühjahr. Die Einschränkungen seit Oktober und der erneute Teil-Lockdown seit November haben die wirtschaftliche Erholung nach dem Lockdown im Frühjahr erst einmal gestoppt. Die in letzter Zeit erlassenen Beschränkungen behindern das Wirtschaftsgeschehen erneut, in einigen Wirtschaftsbereichen sehr massiv. Zudem müssen Tausende in Quarantäne, was auch die Wirtschaftstätigkeit in anderen Bereichen teils einschränkt. Nicht zuletzt wirkt sich das wieder verstärkte Pandemiegeschehen auch auf die Konsum- und auf die Investitionsneigung dämpfend aus. Da fast die ganze Welt und dabei nicht zuletzt unsere europäischen Handelspartner zumeist noch stärker als wir mit der Epidemie kämpfen, wird auch der Außenhandel und damit die deutsche Exportwirtschaft nach zwischenzeitlicher Besserung wieder in Mitleidenschaft gezogen.

In den nächsten Monaten, wenn die Zahl der Infektionen voraussichtlich hoch bleibt und umfangreiche Beschränkungen fortbestehen, wird der Erholungsprozess stocken. Die gesamte Wirtschaftstätigkeit dürfte im Winterhalbjahr kaum mehr zunehmen, sie könnte zunächst sogar wieder etwas zurückgehen. Wenn es nicht zu einem stärkeren Rückschlag im Jahresschlussquartal kommt, dann, weil wegen des Auslaufens der befristeten Mehrwertsteuersenkung der private Konsum noch durch vermehrte Großanschaffungen gestützt werden dürfte. Die durch die hohen Infektionszahlen bedingten Einschränkungen treffen insbesondere das „Alltagsleben" und damit vor allem erneut jene Wirtschaftsbereiche, die schon unter der ersten Welle besonders litten, sodass es hier vermehrt zu Insolvenzen kommen wird. Zudem werden Betriebe, welche die Situation bislang mit Kurzarbeit überbrückten, in zunehmendem Maße auch Entlassungen vornehmen. Die Zahl der Arbeitslosen wird folglich wieder zunehmen. Nach dem starken Aufholprozess im dritten Quartal dürfte das reale BIP im vierten Quartal 2020 stagnieren; im Jahresdurchschnitt 2020 wird es um 5 % niedriger als im Vorjahr sein (vgl. Tabelle 1). Die Verbraucherpreise werden infolge der Mehrwertsteuerreduzierung bis zum Jahresende noch knapp unter der Nulllinie bleiben.

© Der/die Autor(en) 2020. Open Access: Dieser Artikel wird unter der Creative Commons Namensnennung 4.0 International Lizenz (https://creativecommons.org/licenses/by/4.0/deed.de) veröffentlicht.

Open Access wird durch die ZBW – Leibniz-Informationszentrum Wirtschaft gefördert.

Erst im Frühsommer 2021, wenn nach den bisherigen Erfahrungen die Zahl der Infektionen witterungsbedingt wieder zurückgeht und voraussichtlich ein Impfstoff verbreitet verfügbar sein wird, setzt sich mit Rücknahme der Einschränkungen der Erholungsprozess der Wirtschaft fort. Die Inlandsnachfrage und wohl auch die Auslandsnachfrage werden sich dann wieder festigen. Dabei wird dann auch einiges an Konsum (nicht zuletzt an „Freizeitkonsum" wie Reisen und Events) und Investitionen nach-

Abbildung 1

**Preisbereinigtes Bruttoinlandsprodukt in Deutschland**

Saison- und arbeitstäglich bereinigt

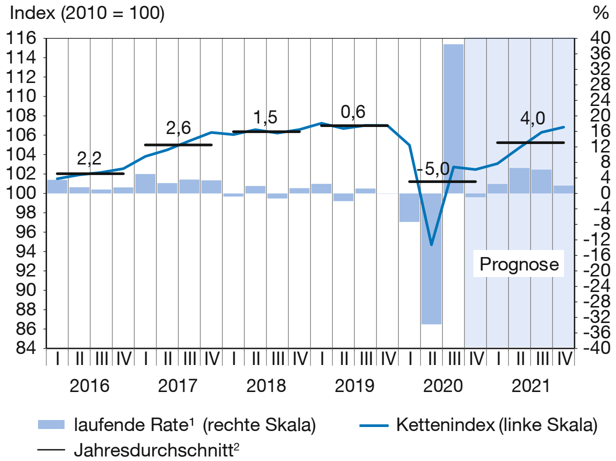

[1] Veränderung gegenüber dem Vorquartal in %, auf Jahresrate hochgerechnet. [2] Zahlenangaben: Veränderung gegenüber dem Vorjahr in %.

Quellen: Statistisches Bundesamt; ab 4. Quartal 2020 Prognose des HWWI.

Tabelle 1
**Eckdaten für Deutschland**

Veränderung gegenüber dem Vorjahr in %

| | 2019 | 2020 | 2021 | 2022 |
|---|---|---|---|---|
| Bruttoinlandsprodukt[1] | 0,6 | -5,0 | 4,0 | 2,5 |
| Private Konsumausgaben | 1,6 | -6,2 | 4,1 | 2,0 |
| Staatliche Konsumausgaben | 2,7 | 3,9 | 0,4 | -1,1 |
| Anlageinvestitionen | 2,5 | -3,6 | 3,8 | 3,4 |
| Ausrüstungen | 0,5 | -12,5 | 7,3 | 5,5 |
| Bauten | 3,8 | 2,0 | 0,9 | 2,2 |
| Sonstige Anlagen | 2,7 | -3,0 | 5,7 | 3,1 |
| Inlandsnachfrage | 1,2 | -3,8 | 3,5 | 2,2 |
| Ausfuhr | 1,0 | -10,3 | 7,7 | 5,4 |
| Einfuhr | 2,6 | -8,5 | 7,2 | 5,0 |
| Arbeitsmarkt | | | | |
| Erwerbstätige | 0,9 | -1,0 | 0,0 | 0,7 |
| Arbeitslose (in Mio.) | 2,27 | 2,72 | 2,82 | 2,58 |
| Arbeitslosenquote[2] (in %) | 4,8 | 5,7 | 5,9 | 5,4 |
| Verbraucherpreise | 1,4 | 0,5 | 1,1 | 1,7 |
| Finanzierungssaldo des Staates (in % des BIP) | 1,5 | -5,1 | -3,1 | -1,0 |
| Leistungsbilanzsaldo[3] (in % des BIP) | 7,2 | 7,1 | 7,2 | 7,3 |

[1] Preisbereinigt. [2] Arbeitslose in % der inländischen Erwerbspersonen (Wohnortkonzept). [3] In der Abgrenzung der Zahlungsbilanzstatistik.

Quellen: Statistisches Bundesamt; Deutsche Bundesbank; Bundesagentur für Arbeit; ab 2020 Prognose des HWWI.

geholt. Die kurzfristigen Wachstumsraten (von Quartal zu Quartal) werden also im Sommerhalbjahr 2021 gegenüber dem Winterhalbjahr wieder deutlich höher ausfallen. Die jahresdurchschnittliche Wachstumsrate 2021 wird wegen des etwas gedrückten Ausgangsniveaus (Überhang) zur Jahreswende 2020/2021 mit 4 % etwas niedriger sein als in den vorangegangenen Prognosen (ohne zweite Welle) angenommen. Die Situation am Arbeitsmarkt wird sich im Jahresverlauf 2021 wieder verbessern. Die Zahl der Arbeitslosen dürfte aber im Jahresdurchschnitt mit 2,8 Mio. noch etwas höher sein als 2020. Die Inflationsrate wird nach Auslaufen der Mehrwertsteuerreduzierung Ende 2020 zu Beginn kommenden Jahres wieder deutlicher ansteigen, aber auch im späteren Jahresverlauf wohl nicht die 2 %-Marke überschreiten.

Im Jahr 2022 sollte eine Rückkehr zur „Normalität“ möglich sein. Davon werden vor allem jene Branchen profitieren, die aktuell besonders unter den coronabedingten Einschränkungen leiden, wie Gastronomie, Hotellerie, Tourismus, Veranstaltungsbranche und Ähnliches. Zudem dürften sich auch die Weltwirtschaft und damit der Welthandel nachhaltig erholen. Mit den verbesserten Perspektiven werden die Unternehmen ihre Investitionen wieder merklich ausweiten. Die Entwicklung der deutschen Wirtschaft sollte sich dann wieder weitgehend dem potenziellen Wachstumspfad nähern. Dank eines noch erheblichen Überhangs aus dem Jahr 2021 wird die gesamtwirtschaftliche Leistung im Jahresdurchschnitt 2022 um 2½ % zunehmen. Die Lage am Arbeitsmarkt dürfte sich weiter entspannen. Der Anstieg der Verbraucherpreise bleibt in der Grundtendenz moderat.

Die Vorausschätzungen stehen weiterhin unter der Prämisse, dass es nicht zu abermaligen Rückschlägen in der Corona-Entwicklung kommt. Zusätzliche Einschränkungen in den kommenden Wintermonaten können im Falle eines weiteren Anstiegs der Infektionszahlen nicht ausgeschlossen werden. Auch zeichnet sich ein harter Brexit ab. Bei sich wieder belebender Weltwirtschaft sollte dieser allerdings insgesamt nur zu marginalen Handelseinbußen führen. Bei der hier erwarteten Entwicklung wird die deutsche Wirtschaft 2022 wieder das Vor-Corona-Niveau erreichen. Gleichwohl bedeutet das gegenüber einer möglichen Entwicklung ohne Corona einen gewissen Wachstumsverlust, zumal sich durch die schwache Investitionsentwicklung während der Corona-Krise auch der Potenzialpfad abgeflacht hat.

Ohne die umfangreichen staatlichen Hilfen wäre allerdings der Einbruch der Wirtschaft noch tiefer und wohl dauerhafter gewesen. Die Zahl der Insolvenzen und die der Arbeitslosen wären dann wesentlich höher ausgefallen und hätten längerfristigere negative Auswirkungen auf das Wirtschaftswachstum gehabt. Sicherlich steigt dadurch die Staatsverschuldung schubartig. Doch ist die tatsächliche Verschuldung niedriger als die Summe der verabschiedeten Hilfsgelder, denn zum einen bedeuten Kredithilfen und Bürgschaften nicht a priori neue Bestandsschulden, zum anderen werden wohl nicht alle Mittel in Anspruch genommen. Zudem wären bei einem tieferen und längeren Einbruch der Wirtschaft künftig die Staatseinnahmen geringer und bestimmte Sozialausgaben höher ausgefallen und hätten dann für höhere Schulden und/oder Einsparungen, auch bei Infrastrukturinvestitionen, geführt. Und letztlich wird in der gegenwärtigen und wohl noch auf Jahre absehbaren Niedrigzinsphase – aktuell sogar Minuszinsen – der Staatshaushalt nicht durch zusätzliche Zinsausgaben belastet. Und zuallerletzt muss der Staat fällig werdende Rückzahlungen nicht unbedingt sofort leisten, sondern kann sie, wie in der Vergangenheit mehr oder weniger üblich, prolongieren. Dabei kommt der deutschen Politik auch ihre in den vergangenen Jahren solide Haushaltspolitik zugute, die seit 2012 für Überschüsse im gesamten Staatshaushalt sorgte und die Staatsschuldenquote (Staatsverschuldung in Relation zum BIP) von damals über 80 % wieder knapp unter die Stabilitätsmarke von 60 % drückte.

Jörg Hinze
j-hinze@hwwi.org
